# Technical Report of Radiofrequency Ablation of AVNRT with Persistent Left Superior Vena Cava: Success Relies on Basics

**DOI:** 10.3390/jcm14072477

**Published:** 2025-04-04

**Authors:** Mohamed A. Elhadad, Ramin Ebrahimi, Gozal Mirzayeva, Anna Neumann, Daniel Schneppe, Sarah Janschel, Márcio Galindo Kiuchi, Piotr Futyma, Helmut Pürerfellner, Shaojie Chen

**Affiliations:** 1 Department of Internal Medicine B (Cardiology, Angiology, Pneumology and Internal Intensive Care Medicine), University Medicine Greifswald, 17475 Greifswald, Germany; 2 School of Medicine-Royal Perth Hospital Unit, University of Western Australia, Perth 6009, Australia; 3 Clinical Electrophysiology, St. Joseph’s Heart Rhythm Center, 35-623 Rzeszów, Poland; 4 Collegium Medicum, University of Rzeszów, 35-310 Rzeszów, Poland; 5 Department of Cardiology/Electrophysiology, Akademisches Lehrkrankenhaus, Ordensklinikum Linz Elisabethinen, 4020 Linz, Austria; 6 Rhythmology and Clinical Cardiac Electrophysiology, Klinik of Internal Medicine B (Cardiology, Angiology, Pneumology and Internal Intensive Care Medicine), University Medicine Greifswald, Ferdinand-Sauerbruch-Straße, 17475 Greifswald, Germany

**Keywords:** AVNRT, persistent left superior vena cava, ablation, atrioventricular nodal reentrant tachycardia, ablation, Koch triangle, slow pathway

## Abstract

**Background/Objectives**: Persistent Left Superior Vena Cava (PLSVC) is a condition that may complicate the ablation of Atrioventricular nodal reentry tachycardia (AVNRT). We aimed to report technical experience in ablation under scuh clinical setting. **Methods**: 3D guided electrophysiological procedure was conducted and PLSVC was confirmed. Slow-pathway ablation for the AVNRT was performed and typical junctional rhythm during the ablation was observed. **Results**: Exactly the same AVNRT remained inducible after 10 radiofrequency applications, which was very likely because of suboptimal temperature increase due to lacking sustained stability/contact of the catheter given the PLSVC anatomy and the patient’s deep respiration based on our observation during the RF applications. A non-steerable long sheath was introduced to achieve more firm contact of the ablation catheter, the slow-pathway was successfully ablated with just 1 application (seen immediately occurred, continuous typical junctional rhythms during ablation, and significantly better temperature during the ablation). **Conclusions**: PLSVC-related anatomical changes may destabilize ablation catheter making it difficult to establish sufficient energy delivery at the slow-pathway region and put forward the need for multiple ablations. Timely identifying such scenarios (e.g., insufficient stability, insufficient temperature) could help better plan/change the ablation technique or strategy to achieve better procedure outcomes. This technical report reminds us that typical junctional beats may not be the only determinant for successful ablation of the slow-pathway. The key to the solution often relies on basic ablation biophysics.

## 1. Introduction

Slow pathway (SP) ablation is a well-established procedure for the treatment of atrioventricular nodal reentrant tachycardia (AVNRT). Identification of the Koch triangle (KT) and localization of the SP potentials to guide an electrogram (EGM)-based ablation remains the classic approach of SP ablation [1,2].

The presence of a Persistent Left Superior Vena Cava (PLSVC) may pose challenges to the ablation procedure because of the variated anatomy, potentially resulting in difficulties during the procedure [3,4,5,6,7,8].

We present an informative case of successful ablation in AVNRT complicated with PLSVC; this case underscores the importance of basic ablation biophysics during ablation of SP in patients with PLSVC.

## 2. Method and Results

### 2.1. Clinical Presentation

A 40-year-old female patient with known history of supraventricular tachycardia and known left bundle branch block since many years, presented to our emergency room with a new episode of palpitations; ECG revealed a regular tachycardia with a heart rate of 165 beat/minute, Valsalva maneuver and beta-blocker did not terminate the tachycardia. Possible causes (e.g., electrolyte disturbance, hyperthyroidism, ischemic heart disease) for arrhythmia had been ruled out. An electrophysiologic study and catheter ablation of the SVT was suggested. The patient was fully informed, and written informed consent was obtained.

### 2.2. Diagnostic Workup

**Figure 1:**
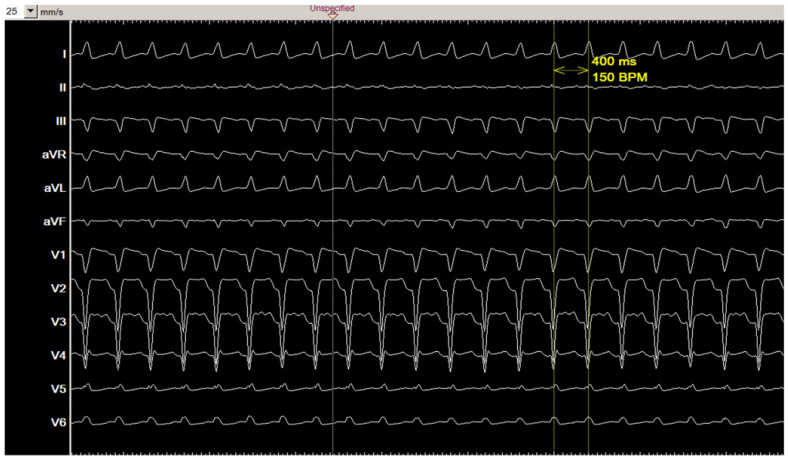
The 12-lead ECG of the clinical tachycardia.

Figure 1 shows the 12-lead ECG of the clinical tachycardia. During the electrophysiological study, diagnostic catheters were positioned in coronary sinus (CS), right ventricle (RV), and at HIS position. Baseline ECG showed sinus rhythm with known left bundle branch block, AH interval: 98 ms, HV interval: 45 ms. 

**Figure 2:**
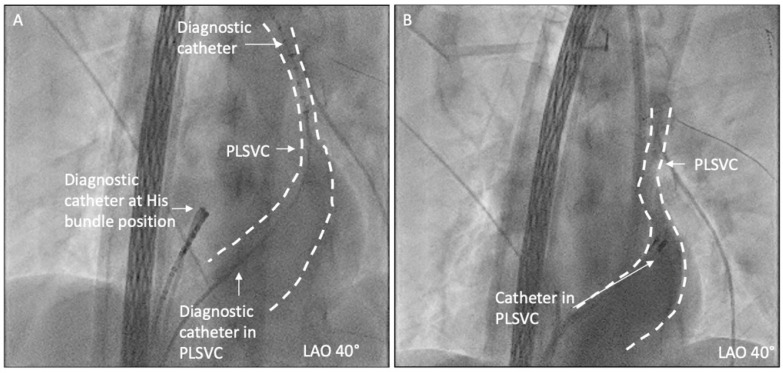
PLSVC was confirmed by fluoroscopy and angiography in CS. (**A**) A diagnostic catheter is positioned in distal PLSVC; (**B**) angiography in CS shows the existence of PLSVC.

During the positioning of the catheter in the CS, existence of PLSVC was highly suspected, and the PLSVC was confirmed by angiography in the CS (as shown in Figure 2).

**Figure 3:**
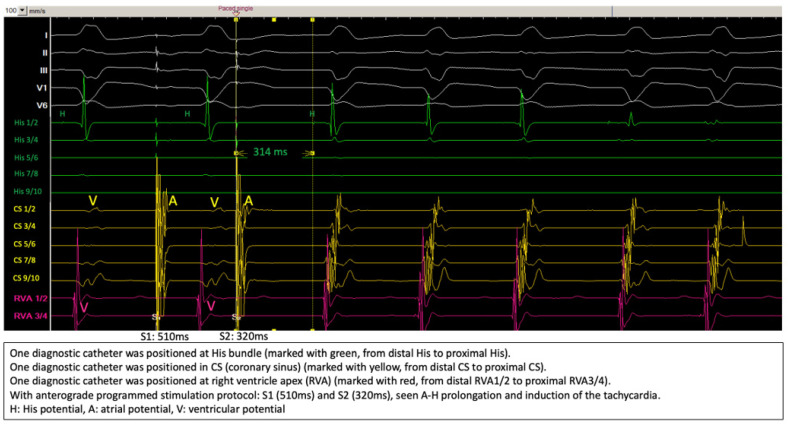
Induction of the tachycardia during electrophysiologic study.

Figure 3 shows the induction of the clinical tachycardia with typical A-H jump phenomenon, the diagnosis of typical slow-fast AVNRT was further verified by differential pacing maneuvers.

### 2.3. Ablation Strategy

**Figure 4:**
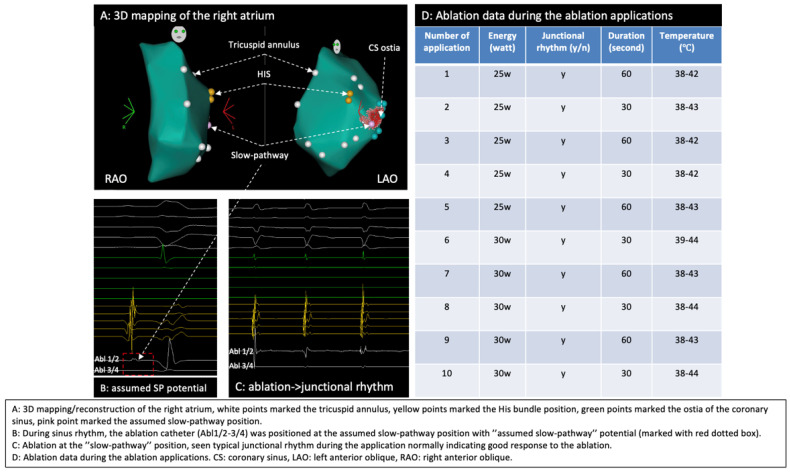
Three-dimensional mapping of the right atrium, identification of the slow pathway, and ablation. (**A**) Three-dimensional mapping of the right atrium; (**B**) SP region marked the SP potential; (**C**) junctional rhythm during ablation; (**D**) ablation data during RF applications.

By using a 3D mapping system (Carto 3, Biosense Webster, Irvine, CA, USA), a 3D anatomical model of the right atrium (RA) was reconstructed where mitral annulus, HIS position, CS and the slow pathway region were marked (Figure 4A), typical slow pathway potential was documented at the slow pathway region (Figure 4B), conventional non-irrigated RF ablation (NAVISTAR^®^ Ablation Catheter, Biosense Webster, Irvine, CA, USA) of the slow pathway for the AVNRT was performed and typical junctional rhythm during the ablation was observed (Figure 4C).

### 2.4. Outcome

**Figure 5:**
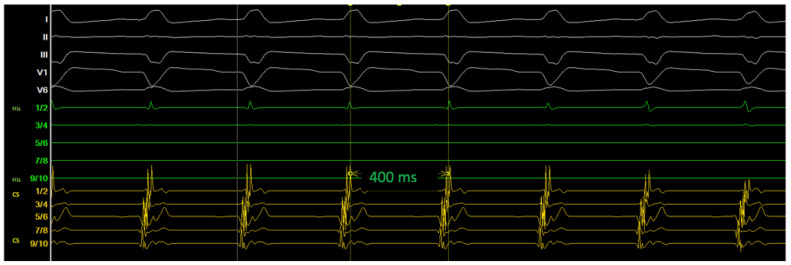
After 10 applications of ablation at the SP region, AVNRT remained inducible, likely due to insufficient catheter contact and instability.

Ablation data during the RF applications are summarized in Figure 4D. Despite intermittent typical junctional rhythm during the ablation, the AVNRT was still inducible (Figure 5) after 10 RF applications, which was very likely because of suboptimal temperature increase due to lacking sustained stability/contact of the catheter given the PLSVC anatomy and the patient’s deep respiration based on our observation during the RF applications. The cycle length of the reinduced AVNRT was 400 ms, supporting the hypothesis that the initial ablations were completely unsuccessful, as the tachycardia was not only inducible but also showed the same cycle length because the slow pathway conduction was not even minimally modified.

**Figure 6:**
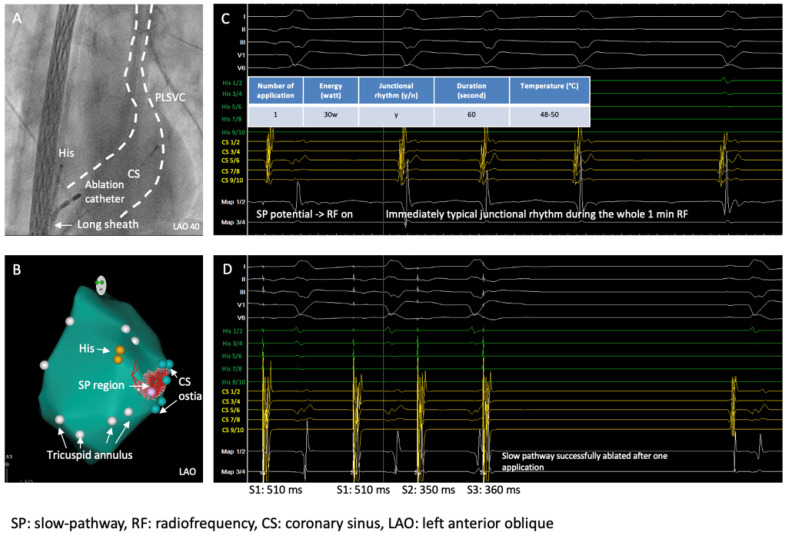
A long sheath used for better catheter contact and catheter stability, and SP was then successfully ablated with one ablation application. (**A**) Fluoroscopy shows the CS, PLSVC, long sheath, and ablation catheter; (**B**) 3D reconstruction of the right atrium, including mitral annulus (white points), His bundle (yellow points), CS ostia (green points), SP region (pink); (**C**) last ablation application at SP region, immediately typical junctional rhythm during the RF application; (**D**) SP successfully ablated with only one 1 RF application.

We then decided to introduce a non-steerable long sheath (SL-1, St. Jude Medical, Saint Paul, MN, USA) to achieve more stable contact of the ablation catheter (Figure 6A), after which we successfully ablated the slow pathway with just 1 application (seen immediately occurred, continuous typical junctional rhythms during ablation, and significantly better temperature, max. 50 °C, during the ablation) (Figure 6B,C).

Repeating EP study after the ablation showed no more induction of the AVNRT and successful elimination of the slow pathway without evidence of AV block (Figure 6D).

Complications were further excluded by echocardiography and continuous in-hospital ECG monitoring. The patient had no evidence (palpitation or ECG documentation) of arrhythmia recurrence during 6M follow-up, and no other cardiac arrhythmias were revealed.

## 3. Discussion

AVNRT is the most common paroxysmal supraventricular tachycardia in clinical practice. Typical slow-fast AVNRT is caused by a re-entry circuit with slow antegrade and fast retrograde pathways of the AV node. PLSVC is the most common thoracic venous anomaly, present in approximately 0.2–0.6% of the general population. Its prevalence increases to 4.5–9% in individuals with congenital heart diseases [8,9].

In a large sample multicenter retrospective study, authors found that patients with PLSVC had a higher incidence of various SVTs compared to the general population. Interestingly, the presence of PLSVC was linked to unique electrophysiological substrates that predispose patients to SVTs. The anatomical and structural characteristics of PLSVC can facilitate the development of arrhythmias. The authors also found among the patients with SVTs associated with PLSVC undergoing catheter ablation, the procedure was effective in eliminating arrhythmias in most cases. However, the study highlighted the need for careful mapping and consideration of the unique anatomy when performing ablation in these patients [8].

The presence of PLSVC can complicate catheter ablation procedures due to its anatomical variations, such as an enlarged coronary sinus, which may obscure standard anatomic targets. This can change the typical catheter positioning and pose challenges during electrophysiological mapping and ablation. PLSVC can alter access to the coronary sinus (CS), which is essential for both mapping the atrioventricular (AV) node and delivering ablation energy; therefore, a more focused mapping around the CS, His bundle region, and surrounding areas is needed.

Ideally, imaging such as echocardiography or thoracic CT would help confirm the presence and define the anatomy of PLSVC. In case of no pre-procedural imaging, using fluoroscopy or intracardiac echocardiography (ICE) during the EP procedure can still help define catheter positioning, the course of PLSVC, and the landmarks of Koch’s triangle. Using ICE in complex anatomical situations during ablation procedures can offer several important benefits, such as improved visualization of cardiac structures, accurate catheter positioning and navigation, reduced need for fluoroscopy, and enhanced safety (i.e., real-time monitoring of possible complications, e.g., pericardial effusion) [10]. In a recent randomized study, the utilization of ICE during AVNRT (SP ablation) procedure demonstrated significantly reduced RF time and procedure time as compared to procedures purely guided by 3D mapping [11]. Nonetheless, 3D mapping is an established technology for the majority of EP procedures nowadays; utilizing a 3D mapping system can still help exactly visualize and tag the anatomical and electrophysiological information.

The identification of PLSVC in this case was first suspected by positioning the diagnostic catheter in the CS and then confirmed by angiography in the CS. On the other hand, TTE is a routine noninvasive examination tool for screening the existence of PLSVC, where usually a large CS by TTE views can be observed.

RF ablation for AVNRT complicated with PLSVC can be difficult because of the significantly enlarged CS ostia, which can result in insufficient contact during RF application. It should be mentioned that the assumption of poor contact was made on circumstantial evidence since no contact force sensing catheter was used. In addition, the higher blood flow from the enlarged coronary sinus ostia could have contributed with a cooling effect to lowering the temperature during ablation in the absence of firm and stable electrode/tissue contact.

Although during the first round of RF applications typical junctional rhythms were seen albeit the temperature during RF fluctuated and was not optimal for effective RF lesion formation. Because AVNRT is a type of supraventricular tachycardia that involves a reentrant circuit (typically a slow pathway and a fast pathway) within or around the atrioventricular node, if the ablation at the slow pathway starts, a slow junctional rhythm during the ablation would normally occur, indicating that the targeted slow pathway at the atrioventricular node is irritated/interrupted by the ablation. In other words, the presence of such junctional rhythm during ablation can help in localizing the desired site of the ablation and be observed as a good response of slow pathway ablation.

After introducing a long sheath for better stabilization of the ablation catheter, we successfully ablated the SP with just 1 application; typical junctional rhythms immediately occurred right after RF delivery and lasted for the whole 60 s RF delivery; notably, the temperature during ablation was substantially higher as previous RF applications (48–50 °C vs. 38–44 °C). Short-term and midterm follow-ups showed further favorable outcomes after the ablation without any arrhythmia recurrence.

There was no complication after ablation in this case; however, it should be mentioned that, as the PLSVC drains into the CS, abnormal catheter manipulation could cause injury to the coronary sinus, potentially resulting in cardiac tamponade. Careful attention should be paid to guide wire, sheath, and catheter placement. It is known that PLSVC may predispose to other cardiac arrhythmias, particularly atrial arrhythmias like atrial fibrillation. It is, therefore, essential to perform regular follow-up after the procedure.

## 4. Summary

**Figure 7:**
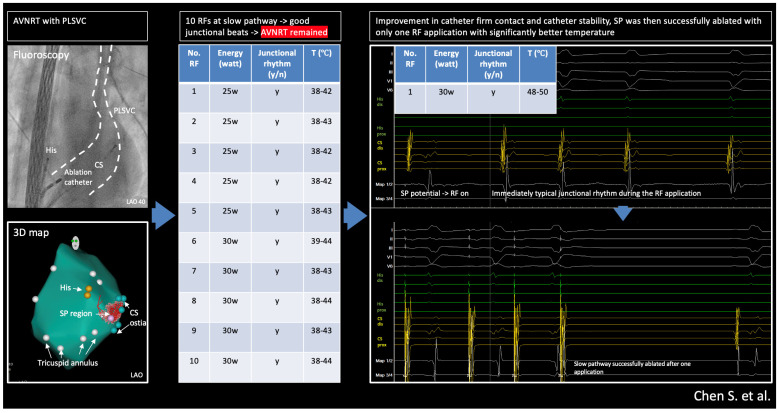
Central illustration: radiofrequency ablation of AVNRT with Persistent Left Superior Vena Cava: success relies on basics.

The central illustration is shown in Figure 7. Persistent Left Superior Vena Cava (PLSVC) is a condition that may complicate the ablation of AVNRT.

PLSVC-related anatomical changes may destabilize the ablation catheter, making it difficult to achieve sufficient energy delivery at the slow-pathway (SP) region and put forward the need for multiple ablations which may cause edema or complications. Timely identifying such scenarios (e.g., insufficient stability or insufficient temperature) could help better plan/change the ablation technique or strategy to achieve better procedure outcomes. This technical report reminds us that typical junctional beats may not be the only determinant for successful ablation of the SP. The key to the solution often relies on basic ablation biophysics.

## Data Availability

Data are contained within the article.
